# Noninvasive Prenatal Paternity Testing (NIPAT) through Maternal Plasma DNA Sequencing: A Pilot Study

**DOI:** 10.1371/journal.pone.0159385

**Published:** 2016-09-15

**Authors:** Haojun Jiang, Yifan Xie, Xuchao Li, Huijuan Ge, Yongqiang Deng, Haofang Mu, Xiaoli Feng, Lu Yin, Zhou Du, Fang Chen, Nongyue He

**Affiliations:** 1 State Key Laboratory of Bioelectronics, School of Biological Science and Medical Engineering, Southeast University, Nanjing, 210096, China; 2 BGI Education Center, University of Chinese Academy of Sciences, Beijing, 100049, China; 3 BGI-Shenzhen, Shenzhen, 518000, China; 4 Department of stomatology, The Second People’s Hospital of Shenzhen, Shenzhen, 518000, China; 5 Center of Forensic Sciences, Beijing Genomics Institute, Beijing, 100049, China; 6 Public Security Bureau of Shenzhen Municipality, Shenzhen, 518000, China; 7 Joint Laboratory of Gene-associated Application Research in Forensics, Shenzhen, 518000, China; 8 Shenzhen Municipal Key Laboratory of Birth Defects Screening and Engineering, BGI-Shenzhen, Shenzhen, 518000, China; 9 Guangdong Provincial Key Laboratory of human diseases genome, BGI-Shenzhen, Shenzhen, 518000, China; 10 Section of Molecular Disease Biology, Department of Veterinary Disease Biology, Faculty of Health and Medical Sciences, University of Copenhagen, Copenhagen, 1165 København K, Denmark; National Cheng Kung University College of Medicine, TAIWAN

## Abstract

Short tandem repeats (STRs) and single nucleotide polymorphisms (SNPs) have been already used to perform noninvasive prenatal paternity testing from maternal plasma DNA. The frequently used technologies were PCR followed by capillary electrophoresis and SNP typing array, respectively. Here, we developed a noninvasive prenatal paternity testing (NIPAT) based on SNP typing with maternal plasma DNA sequencing. We evaluated the influence factors (minor allele frequency (MAF), the number of total SNP, fetal fraction and effective sequencing depth) and designed three different selective SNP panels in order to verify the performance in clinical cases. Combining targeted deep sequencing of selective SNP and informative bioinformatics pipeline, we calculated the combined paternity index (CPI) of 17 cases to determine paternity. Sequencing-based NIPAT results fully agreed with invasive prenatal paternity test using STR multiplex system. Our study here proved that the maternal plasma DNA sequencing-based technology is feasible and accurate in determining paternity, which may provide an alternative in forensic application in the future.

## Introduction

The discovery of cell-free fetal DNA (cffDNA) in maternal blood in 1997 provides the possibility to develop novel noninvasive prenatal paternity testing, which can avoid the procedure-associated fetal loss as well as the restriction of sampling time [1]. In recent years, a few research groups reported pilot studies about noninvasive genotyping of maternal plasma DNA using short tandem repeat (STR) and single-nucleotide polymorphisms (SNP) for paternity determination. In 2009, Jasenka et al. first developed a method based on capillary electrophoresis (CE) detection of STR markers in maternal plasma DNA for noninvasive prenatal paternity test [2]. In their study, only 1–6 informative autosomal STR loci were obtained in 20 pregnancies (13 with male fetus and 7 with female fetus) and 6–16 Y-STR loci could be observed in 13 pregnancies with male fetus. Without enough effective STR loci, it was hard to make a trustful paternity determination. Then in 2011, Tynan et al. used SNP genotype based method to obtain 5–20 paternal alleles in plasma DNA from 154 pregnancies and provided a potential use for noninvasive prenatal paternity testing [3]. In 2012, Guo et al. used allele-specific PCR to observe the difference of biological father and unrelated man and describe the basic requirement of informative SNP for paternity exclusion [4]. In 2013, Ou et al. used methylation-sensitive restriction enzyme to enrich the fetal DNA at rs4688725 and suggested that more effective marker should be selected for paternity testing in the future [5, 6]. In 2013, Ryan et al. used HumanCytoSNP-12 array chip (~ 300,000 SNPs) to perform genotyping of paternal alleles in maternal plasma DNA and set up the normal distribution of 1821 unrelated males [7]. With p-value < 0.0001, 20 out of 21 pregnancies were successfully determined and only one case undetermined.

Here, we combined massively parallel sequencing and SNP-based method together and developed a novel sequencing-based noninvasive paternity testing (NIPAT). We performed systematic evaluation of influencing factors, such as the SNP frequency, required number of effective SNP, sequencing depth, threshold of fetal fraction, as well as sequencing strategy. We validated the reliability of this sequencing-based NIPAT in real clinical samples, thus showing the feasibility of using NIPAT in a clinical setting.

## Materials and Methods

### General study design

**Fig 1:**
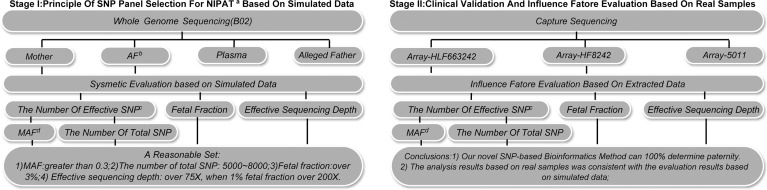
The study workflow. There were two stages in this study. The first stage determined the criteria of SNP panel selection based on the evaluation results from simulation data. The second stage was clinical validation in 16 real pedigrees using three different selective SNP panels.

We designed a two-stage study to evaluate the performance of the noninvasive prenatal paternity testing (NIPAT) based on maternal plasma DNA sequencing. In Phase I, one case was recruited to initiate methodology development and described the characteristics such as several affecting factors. In Phase II, 16 clinical pregnancies were recruited to optimize and validate the feasibility in selected SNP panels and established the quality control system. Finally, we chose the best panel for further validation in real clinical pregnancies. An overview of the study workflow is showed in Fig 1.

### Sample collection and parenthood determination

Seventeen families were recruited from the collaborative hospitals. The gestational age (GA) ranged from 13 to 30^+6^ weeks and the maternal age from 26 to 44 years old. Two-milliliter peripheral blood samples of pregnant women and their husbands were collected into EDTA-containing tubes. Maternal plasma was isolated with a two-step centrifugation protocol [8]. Amniocentesis was performed at 18-20^th^ weeks and five milliliters amniotic fluid was obtained from the hospitals. 100 microliters blood samples from both parents and 500 microliters amniotic fluid from 5 families were sent to Shenzhen Municipal Public Security Bureau (Shenzhen, China) for conventional prenatal paternity test using the AmpFlSTR^®^Identifiler^®^ PCR Amplification Kit (ABI). Meanwhile, sequencing data of 90 unrelated healthy Han Chinese were downloaded from the 1000 Genomes Project (BioProject ID:298320). Detailed pedigree information of all families is listed in Table A in S3 File.

Informed written consent was obtained from each participant. This study was approved by the institutional review board of BGI-Shenzhen and conducted in accordance with the Declaration of Helsinki.

### DNA extraction, Library preparation

Genomic DNA (g-DNA) of parents and amniotic fluids was extracted with QIAamp DNA Mini Kit (Qiagen) following the manufacturer’s instructions. One microgram g-DNA was fragmented by sonication (Covaris). CffDNA in maternal plasma was extracted from 200-microliter maternal plasma by TIANamp Micro DNA Kit (Tiagen) and the DNA was already fragmented by nature. After end blunted, all fragments were added an “A” tail for the ligation with adaptors. Barcodes were introduced during PCR for multiplex sequencing.

Three customer-designed panels were obtained from NimbleGen (Roche). DNA libraries were measured with Agilent 2100 bioanalyzer (Agilent) for insert size and quantified by real-time PCR. Three microgram DNA libraries were hybridized to the SNP panels according to the manufacturer’s instructions [9]. Captured DNA libraries were conducted using 90bp paired-end index sequencing on Hiseq^TM^ 2000 (Illumina) according to the manufacturer’s instructions.

### Reads alignment and SNP calling

The paired-end sequencing reads were mapped to the human reference genome (Hg19, GRCh37) using SOAP2 [10]. The reads that mapped to multiple locations and the duplication reads were removed. SNP calling was performed using the SOAPsnp software in the target region or whole-genome wide [11]. The filter criteria (coverage greater than 8 and quality value higher than 20) were set to guarantee the accuracy of the genomic genotype. Meanwhile, error rate was recorded for quality control purpose before CPI calculation.

When the locus was homozygous in both parents with the same genotypes, we defined error as the situation that the fetal genotype was unexpected heterozygote based on Mendel’s law. The formula $f_{error}=\frac{d_{error}}{d_{mother}+d_{error}}$ was used to calculate the error rate in maternal plasma (f_error_), where d_mother_ and d_error_ stand for the depth of allele from mother and error (all other alleles which was different from mother’s genotype) respectively.

### Estimation of fetal fraction

For locus homozygous in both parents but with different genotypes, the fetal genotype was an obligate heterozygote based on Mendel’s laws. Thus fetal fraction was calculated using the formula $f=\frac{2d_{father}}{d_{mother}+d_{father}}$, where *d*_*father*_ and *d*_*mother*_ stand for the depth of allele from father and mother respectively.

### Mathematical model of SNP-based paternity test

We developed a novel algorithm to describe the paternity index (PI) of a male candidate in a random population based on the effective SNP (the SNP homozygous in mother) in maternal plasma sequencing data. This value was defined as the odds ratio in the formula: $PI=\frac{X}{Y}$. In this formula, *X* = Pr(*Plasma*|*Mother*,*T*), which stands for the probability that male candidate is the biological father; and *Y* = Pr(*Plasma*|*Mother*,*R*), which stands for the probability of that a random man is the biological father. According to the Bayesian model, the PI was calculated as:

$$\begin{array}{l} PI=\frac{X}{Y}=\frac{\mathrm{Pr}(Plasma|Mother,T)}{\mathrm{Pr}(Plasma|Mother,R)}\\ =\frac{\mathrm{Pr}(Plasma,Fetus|Mother,T)}{\mathrm{Pr}(Plasma,Fetus|Mother,R)}\\ =\frac{\sum_{Fetus}\mathrm{Pr}(Fetus|Mother,T)•\mathrm{Pr}(Plasma,Fetus|Mother,Fetus,T)}{\sum_{Fetus}\mathrm{Pr}(Fetus|Mother,R)•\mathrm{Pr}(Plasma,Fetus|Mother,Fetus,R)} \end{array}$$

Thus the combined paternity index (CPI) was calculated as the product of PI: $CPI=\prod_{i=1}PI_{i}$

Moreover, the probability of every candidate fetal genotype (Pr(*Fetus*|*Mother*,*Father*)) was calculated by the Mendel’s Law and independent assortment. The probability of the depth distribution in maternal plasma corresponding to different combination of mother and fetus was calculated by quadrinomial distribution, described as:

$$\mathrm{Pr}(Plasma,Fetus|Mother,Fetus,Father)=\frac{n!}{a_{A}!a_{T}!a_{C}!a_{G}!}p_{A}^{a_{A}}p_{T}^{a_{T}}p_{C}^{a_{C}}p_{G}^{a_{G}}$$

Where *a*_*X*_ means the effective sequencing depth of base X; *p*_*X*_ means the incidence rate of base X, obtained from theoretical probability of occurrence. Additionally, we performed test statistic with 90 unrelated individuals and calculated the p-value [7, 12]. We defined that when the logarithm of CPI (Lg(CPI)) was greater than 4 and p-value<10^−4^, the alleged father was classified as biological father.

## Results

### Systematic evaluation of influence factors to NIPAT

First, we evaluated the influence of MAF to sequencing-based NIPAT. In our bioinformatics pipeline, the binomial distribution was suited to the frequency distribution of the effective SNP in NIPAT. According to the binomial distribution probability model, there was a positive correlation between MAF, the number of total SNP and the number of effective SNP (S1 Fig). To further validate the theory, WGS was performed in S01 (effective sequencing depth of 37.37X) and the obtained SNPs were divided into two groups, namely the high frequency (HF, refers to MAF>0.3 on db135) and low frequency (LF, refers to MAF<0.3 on db135). Although the number of effective SNPs in LF was 1.72 times more than effective SNPs in HF (1.95×10^6^ vs 1.1336×10^6^) in WGS data of S01 plasma, the calculated Lg(CPI) were 9.88×10^4^ and -8.53×10^4^, the error rate were 3.79‰ and 3.20‰, respectively. This preliminary result based showed that for sequencing-based NIPAT, only HF SNPs worked well with not enough deep sequencing depth (40.06X for HF SNPs and 34.68X for LF SNPs). This outcome was consistent with previous study of invasive paternity test [13].

Based on above results, HF SNPs were chosen for NIPAT. To evaluate the other factors affecting NIPAT, simulating sequencing data with consistent error rate was generated using the sequencing data of S01 (maternal g-DNA and amniotic fluid g-DNA). These simulation data had three situations: 1) with 75X sequencing depth, the number of effective SNPs ranged from 10 ~1×10^5^, and the fetal fraction from 1% to 30%; 2) with 10% fetal fraction, the number of effective SNP ranged from 10~1×10^5^ and the effective sequencing depth from 10X~2×10^3^X; 3) with 1×10^3^ effective SNPs, the fetal fraction ranged from 1%-30% and the effective sequencing depth from 10X~2×10^3^X.

**Fig 2:**
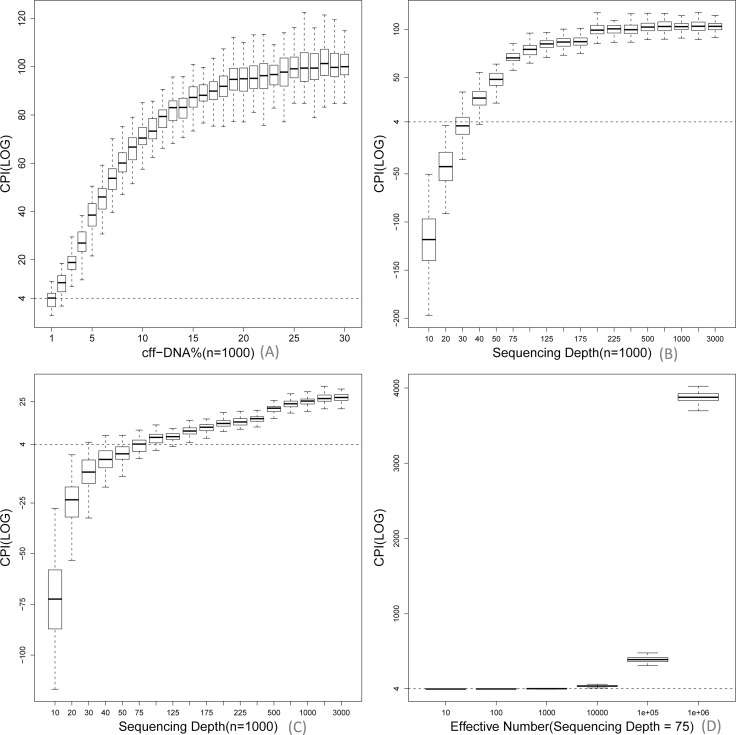
Systematic evaluation of influence factors to sequencing-based NIPAT. (A) In given conditions (1000 effective SNPs and 75X sequencing depth), when the fetal fraction increased from 1% to 10%, the CPI increased dramatically; while once fetal fraction reached to 10%, the calculated CPI increased slightly. (B) In given conditions (1000 effective SNPs, 10% fetal fraction), the initial effective sequencing depth changed from 10X to 75X resulted in a dramatic increase of calculated CPI, whereas the following effective sequencing depth change only brought week increase of calculated CPI, and stay stable when the sequencing depth was over 200X. (C) In special conditions (1% fetal fraction, 1000 effective SNPs), deep sequencing (>125X) was recommend for NIPAT. (D) In special conditions (1% fetal fraction, 75X sequencing depth), a larger number of effective SNPs (>10000) was recommend for NIPAT.

First, the simulated data with the effective sequencing depth of 75X was generated to evaluate the effect of fetal fraction. With the fetal fraction increasing from 1% to 30%, the calculated Lg(CPI) increased from 3.34 (-1.52~-10.74) to 91.84 (30.79~157.30) when the number of effective SNP was 1×10^3^, thus showing significant positive correlation between calculated CPI and fetal fraction. Fetal fraction showed considerable influence to CPI when changed from 1% to 10%. However, once above 10%, fetal fraction had little effect to the calculated CPI (Fig 2A). With the effective sequencing depth of 75X, the samples with fetal fraction less than 3% were applicable for sequencing-based NIPAT when the number of effective SNP number was over 1×10^5^ (S2 Fig).

Second, we evaluated the effect of sequencing depth at the fixed fetal fraction of 10%. With effective sequencing depth increasing from 10X to 200X, the calculated Lg(CPI) increased from -113.66 (-239.24~-21.91) to 90.95 (36.97~150.90) when the number of effective SNP was 1×10^3^, showing a strong positive correlation. The change of effective sequencing depth from 10X to 75X brought obvious improvement of calculated CPI, whereas further increase of effective sequencing depth only slightly improved the calculated CPI. In particular, sequencing depth over 200X did not improve the calculated CPI any more (Fig 2B). Additionally, we observed that the calculated Lg(CPI) was below zero and the determination of paternity was incorrect if the sequencing depth dropped below 30X, no matter how many effective SNPs were used (S3 Fig). For low fetal fraction (~1%) samples, deep sequencing (>125X) or increased number of effective SNP (N>1×10^5^) was recommend (Fig 2C and 2D).

**Fig 3:**
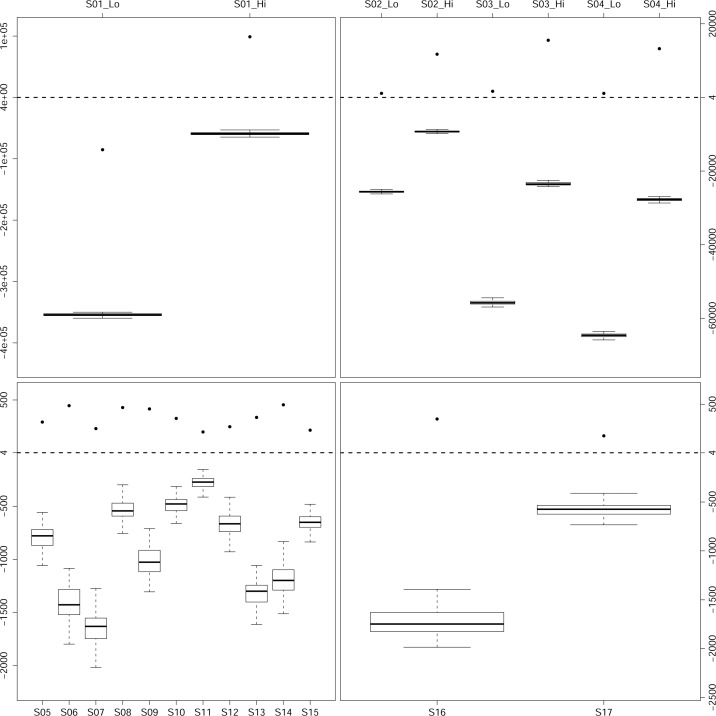
Combined Paternity Index (CPI) in biologic father and random men. The x-axis stands for the sample ID in the top and the bottom; the y-axis marks the logarithmic value of CPI in the left and the right. The logarithm of CPI calculated from 17 real samples were over four, and had a significant separate distribution of CPIs between the biological father and 90 unrelated individuals.

Based on above results, the following conditions were determined for sequencing-based NIPAT: 1×10^3^~2×10^3^ effective SNPs (5×10^3^−8×10^3^ total SNPs in designed panel), MAF of the SNPs greater than 0.3, sequencing depth over 75X, and fetal fraction over 3%. In the condition of fetal fraction less than 3%, >125X sequencing depth was recommended. We firstly performed our bioinformatics pipeline in S01 plasma WGS data to validate the applicability of this method. The calculated Lg(CPI) for NIPAT based on our bioinformatics pipeline in S01 by using effective HF SNP was 9.88×10^4^ and the p-value was < 10^−4^. Meanwhile, the calculated Lg(CPI)for amniotic fluid was 2.92×10^4^ and the p-value was < 10^−4^. With Lg(CPI) > 4 and p-value <0.01, we defined the S01 alleged father as biological father, which was consistent with the results based on invasive paternity test (Fig 3).

### Clinical validation and influence factors evaluation using real sequencing data

#### Clinical validation in three customer-designed SNP panels

Based on simulation results, three customer-designed SNP panels were obtained from NimbleGen (Roche) for further validation of our sequencing-based NIPAT method in clinical samples. Array-HLF663243 contained 3.330×10^6^ LF and 6.63×10^5^ HF SNPs; Array-HF8242 included 8.24×10^3^ HF SNPs, and Array-HF5011 contained 5.01×10^3^ HF SNPs.

The sequencing depth of g-DNA and cffDNA of each family was 26~350 folds and 40–400 folds respectively (Table B in S3 File). The number of theoretically informative SNPs accounted for about 40% of total SNPs in each selective SNP panels (Table B in S3 File). The calculated fetal fraction were 16.65% (7.83~29.74%) in 16 plasma samples (Table 1). The calculated Lg(CPI) for NIPAT based on our bioinformatics algorithm for the biological father and unrelated males were 2.7888×10^3^ (176.78~1.55×10^4^) and -4.5534×10^3^ (-2.87×10^4^~ -153.72) respectively. 11.6% (4.2%~15.4%) of effective SNPs assigned wrongly from the pool of potentially informative SNPs for a tangible parents (Table C in S3 File). The error rate for the biological father and unrelated males were 3.76‰ (2.08‰~5.28‰) and 10.94‰ (5.57‰~28.78‰), respectively. The p-value for the alleged father was all < 10^−4^ (Table 1). We observed significant separation of distribution of CPIs and UHM error between the biological father and unrelated males (Fig 3), suggesting the successful identification of the correct biological father.

**Table 1:** The results of noninvasive prenatal paternity test.

Sample	Fetal fraction	Error rate in plasma	Error rate in AF^1^	Lg(CPI)^2^(Plasma)		NIPAT^3^ Results		Lg(CPI)^2^(AF^1^)	Conventional Paternitytesting results	
				LF^4^	HF^5^	Paternity Inclusion	p-value	HF^5^	CPI^6^	Paternity Inclusion
S01	5.84%	0.38%	0.30%	-8.5256×10^4^	9.8774×10^4^	Yes	<0.0001	2.9172×10^4^	/	Yes
S02	7.83%	0.35%	0.40%	3.9466×10^3^	1.1741×10^4^	Yes	<0.0001	2.6583×10^4^	/	Yes
S03	14.45%	0.30%	0.31%	6.4066×10^3^	1.5523×10^4^	Yes	<0.0001	2.7091×10^4^	/	Yes
S04	14.26%	0.31%	0.44%	3.4925×10^3^	1.3236×10^4^	Yes	<0.0001	2.7330×10^4^	/	Yes
S05	14.86%	0.49%	0.20%	/	292.29	Yes	<0.0001	768.28	1.858×10^7^	Yes
S06	20.21%	0.51%	0.21%	/	446.55	Yes	<0.0001	584.22	8.6138×10^6^	Yes
S07	23.97%	0.53%	0.15%	/	230.99	Yes	<0.0001	549.33	2.9538×10^7^	Yes
S08	11.52%	0.50%	0.20%	/	428.71	Yes	<0.0001	621.84	6.6091×10^7^	Yes
S09	17.55%	0.35%	0.19%	/	416.1	Yes	<0.0001	623.85	/	Yes
S10	10.44%	0.25%	0.16%	/	326.4	Yes	<0.0001	569.62	/	Yes
S11	9.34%	0.37%	0.14%	/	199.22	Yes	<0.0001	536.24	/	Yes
S12	16.52%	0.35%	0.16%	/	248.57	Yes	<0.0001	406.28	/	Yes
S13	18.86%	0.42%	0.22%	/	335.93	Yes	<0.0001	832.35	/	Yes
S14	17.93%	0.31%	0.21%	/	454.49	Yes	<0.0001	746.78	/	Yes
S15	15.78%	0.41%	0.20%	/	215.84	Yes	<0.0001	427.99	/	Yes
S16	29.74%	0.21%	0.13%	/	348.83	Yes	<0.0001	364.06	/	Yes
S17	23.16%	0.36%	0.27%	/	176.78	Yes	<0.0001	195.28	9.0089×10^5^	Yes

1:AF: amniotic fluid

2:Lg(CPI): the logarithm of Combined Paternity Index

3:NIPAT:noninvasive prenatal paternity testing

4:LF:Minor Allele Frequency of SNP <0.3

5:HF:Minor Allele Frequency of SNP >0.32

6:CPI:Combined Paternity Index

To verify our results of paternity decision in NIPAT, we performed our bioinformatics method based on amniotic fluid sequencing data of each family. The calculated Lg(CPI) of the biological father and unrelated males were 5.51×10^3^ (195.28~2.73×10^4^) and -6.31×10^4^ (-3.39×10^5^~-1.19×10^4^) respectively. The error rate for biological father and unrelated males were 2.29‰ (1.33‰~4.36‰) and 41.55‰ (6.27‰~93.89‰), respectively (Table 1). Interestingly, the number of effective SNPs in NIPAT from plasma DNA sequencing and AF DNA sequencing was similar, 1.756×10^4^ (818~9.17×10^4^) and 1.97×10^4^ (872~1.04×10^4^), respectively (Table D in S3 File). We showed that maternal plasma sequencing-based NIPAT could obtained 93.546% (86.21~98.82%) of total effective SNPs in maternal plasma DNA to calculate CPI and determine paternity, which means that sequencing-based NIPAT can obtain similar number of effective SNPs from plasma as from amniotic fluid. Furthermore, the number of effective SNPs had a positive relationship with the number of total SNP in designed array (Table 1), which was consistent with the evaluation results above (S1 Fig).

Seventeen cases underwent sequencing-based paternity test using fetal DNA from amniotic fluid cells. The NIPAT results were 100% (17/17) consistent with the results from invasive paternity test based on amniotic fluid sequencing data. Additionally, 5 in total of 17 cases had conventional paternity test by CE STR, and also showed 100% consistency to NIPAT results (5/5) (Table 1).

#### Influence factors evaluation using real sequencing data

Sequencing data from ten families tested by Array-HF8242 was used to study the four influencing factors to verify the findings from simulation data. We extracted sequencing data from this real clinical plasma sequencing data based on controlling variables method.

**Fig 4:**
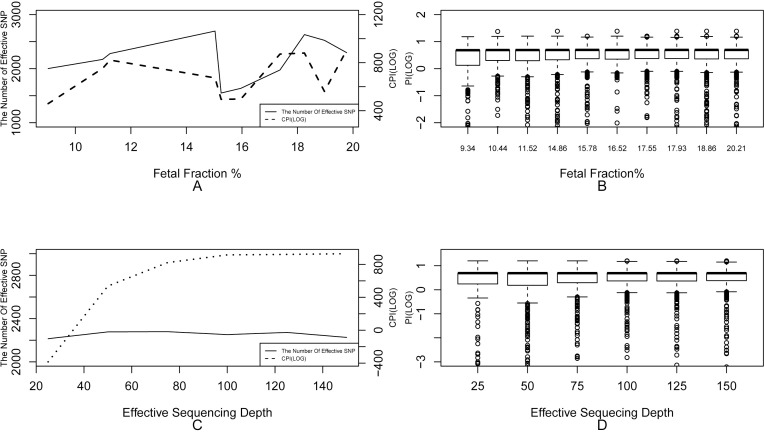
Influence factors evaluation to Combined Paternity Index (CPI) based on real sample sequencing data. (A) The number of effective SNPs and the logarithm of CPI did not relate with the fetal fraction when fetal fraction over 10%. (B) There was no obvious correlation between Paternity Index of each effective SNPs and fetal fraction. (C) The logarithm of CPI had a significant positive correlation with effective sequencing depth, while the number of effective SNPs did not relate with effective sequencing depth. (D) The logarithm of PI had a positive correlation with the effective sequencing depth.

First, we evaluated the influence of the number of effective SNP with fixed effective sequencing depth (75X). There was no significant correlation between CPI or the number of effective SNPs and the fetal fraction with the same effective sequencing depth (75X) and fetal fraction>10% (Fig 4A and 4B, Table E in S3 File). Second, with the fixed number of total SNPs, there was no obvious correlation between the effective sequencing depth and the number of effective SNPs. However, the calculated Lg(CPI) increased from -166.94 to 403.62 with the effective sequencing depth increasing from 25X to 150X (Table E in S3 File). Notably, the calculated Lg(CPI) had no marked increase when the effective sequencing depth reached 75X (Fig 4C and 4D). All above analysis results were consistent with the initial simulation data. Unfortunately, only one family had fetal fraction lower than 10%. To verify the NIPAT performance at low fetal fraction in real clinical case, we extracted data from S01 plasma (sequencing depth = 40X, fetal fraction = 5.84% and the number of effective SNP from 10~1×10^8^). We observed that when effective sequencing depth was relative low (40X), the number of effective SNPs should be larger than 1×10^5^ for a highly accuracy (>99.99%) performance of NIPAT (S4 Fig). This result was consistent with previous evaluated results based on simulating data, which was generated from the mother g-DNA and amniotic fluid genomic sequencing data.

## Discussion

Current STR-based paternity testing was applied for families with postpartum children (vinous blood, saliva, hair, et al.) as well as prenatal fetus (CVS, Amniotic fluid and cord blood). For special cases of prenatal testing, such as cases before the 8th week of pregnancy or contraindicated patients with invasive procedures, a noninvasive prenatal paternity test would be useful and necessary to give out a result. Since plasma cffDNA is fragmented, it is hard to obtain sufficient effective loci of short tandem repeat (STR) to determine paternity by using commercial STR typing kit, especially in female fetus[2]. Considering this, SNP-based noninvasive paternity test could give more reliable information than STR-based method to calculate the combined paternity index (CPI). The advantages of SNP-based noninvasive paternity test include: 1) applicable to short DNA fragments, 2) vast number of SNPs across the whole human genome for analysis. Although a SNP-based method using a high-throughput SNP genotyping array (HumanCytoSNP-12 array chip, ~ 300,000 SNPs) with maternal plasma DNA has been reported for noninvasive paternity testing with high accuracy, a large amount of maternal cffDNA (10mL plasma) was needed in order to get low signal-to-noise genotyping results and aggregating sufficient effective SNPs to do paternity test[7]. Considering the limited probes at each site of genotyping array(~30X) in Ryan’s study, it would miss several father-originated alleles when fetal fraction is lower than 3% and it is impossible to get sufficient effective SNPs in these cases for noninvasive paternity determination[7]. Moreover, the influencing factors, which affect the accuracy of noninvasive paternity testing, such as the allelic frequency of the selected SNPs, the minimal number of total SNPs and the threshold of fetal fraction, remained unevaluated.

Here, we developed a novel, robust and highly accurate maternal plasma DNA sequencing-based noninvasive prenatal paternity testing (NIPAT) and successfully determined paternity in 17 real clinical cases. Furthermore, we evaluated the influence of the number of effective SNPs (total SNPs in designed array), the MAF of SNPs, fetal fraction as well as effective sequencing depth. We observed that the number of effective SNPs had a significant relationship with MAF and the number of total SNPs. In addition, there are individual differences between pregnant women, so the effective SNPs was not similar even using the same capture array. Meanwhile, we systematically evaluated the influence of fetal fraction and effective sequencing depth to the power of NIPAT. Based on our results, we suggested that for NIPAT, the general design should be selecting SNPs with HF MAF of 0.3~0.5, the total number of HF SNP of 5×10^3^~8×10^3^, the sequencing depth 75-200X, which could reach over highly accuracy (>99.9999% based on the 50,400 simulated samples) by using maternal plasma. There is a need for perform noninvasive paternity test in first trimester, however the low fetal fraction (<3.5%) made it difficult to accurate determine paternity. We simulated conditions with 1% fetal fraction, 8×10^3^ total SNPs and 200X effective sequencing depth, and we accurately determined paternity from maternal plasma, providing basic data for NIPAT in first trimester by using our bioinformatics method.

There were some limitations in our study. First, all our recruited samples were in the second trimester (12-20^th^ week) with relatively high fetal fraction (5.68%~29.74%). Further study should be performed with samples in the first trimester (<12^th^ week) with lower fetal fraction. Second, the SNP was selected from db135 with MAF >0.3, validated only in Chinese population. Other ethnic groups need to be verified in the subsequent study. Last, a large-scale study should be developed to evaluate the accuracy of this method in clinical samples.

Here we described a proof-of-concept study of a novel SNP-based NIPAT through maternal plasma DNA sequencing, which showed high accuracy in real clinical cases and may provide an alternative in the application of noninvasive prenatal paternity testing in first trimester in the future.

## Supporting Information

S1 Fig Influence of MAF and the number of total SNPs to the number of effective SNP. The number of effective SNPs had a positive correlation with the MAF and the number of total SNPs in designed array.
(TIF)

S2 Fig Influence of the cffDNA concentration and number of effective loci in the SNP-based paternity test. In the boxplots, the y-axis marks the logarithmic value of combined paternity index, and the x-axis stands for the concentration of cffDNA in the plasma. The plots of a, b, c, d, e and f are corresponded to different number of effective loci, which is predicted in the x-axis.
(TIF)

S3 Fig Influence of the sequencing depth in the SNP-based paternity test. In the boxplots, the y-axis marks the logarithmic value of CPI, and the x-axis stands for the sequencing depth of the plasma. The plots of a, b, c, d, e and f are corresponded to different number of effective loci, which is predicted in the x-axis.
(TIF)

S4 Fig Influence of the number of effective SNPs in the low fetal fraction sample (S01, effective sequencing depth = 40X). In the boxplots, the y-axis marks the logarithmic value of combined paternity index, and the x-axis stands for the number of effective SNPs.
(TIF)

S1 File Formula detail information. Detail information of PI calculation formula was listed in the S1 file.
(DOCX)

S2 File Loci information of three arrays. Detail loci information of three arrays was listed in the S2 file.
(RAR)

S3 File Supporting tables. All supporting Tables were listed in the S3 File. Table A listed the clinical information of all samples. Table B listed the basic sequencing information of all samples. Table C listed the number of SNPs where assigned wrongly from the pool of potentially informative SNPs for a tangible parents. Table D listed the information of effective depth and the number of effective SNP of all samples. Table E listed the basic simulated information of clinical samples.
(XLSX)
