# Supplementary figures and images for: Noninvasive Prenatal Paternity Testing (NIPAT) through Maternal Plasma DNA Sequencing: A Pilot Study
Source: PLoS One. 2016 Sep 15;11(9):e0159385. doi: 10.1371/journal.pone.0159385 (PMC5025199; doi:10.1371/journal.pone.0159385)

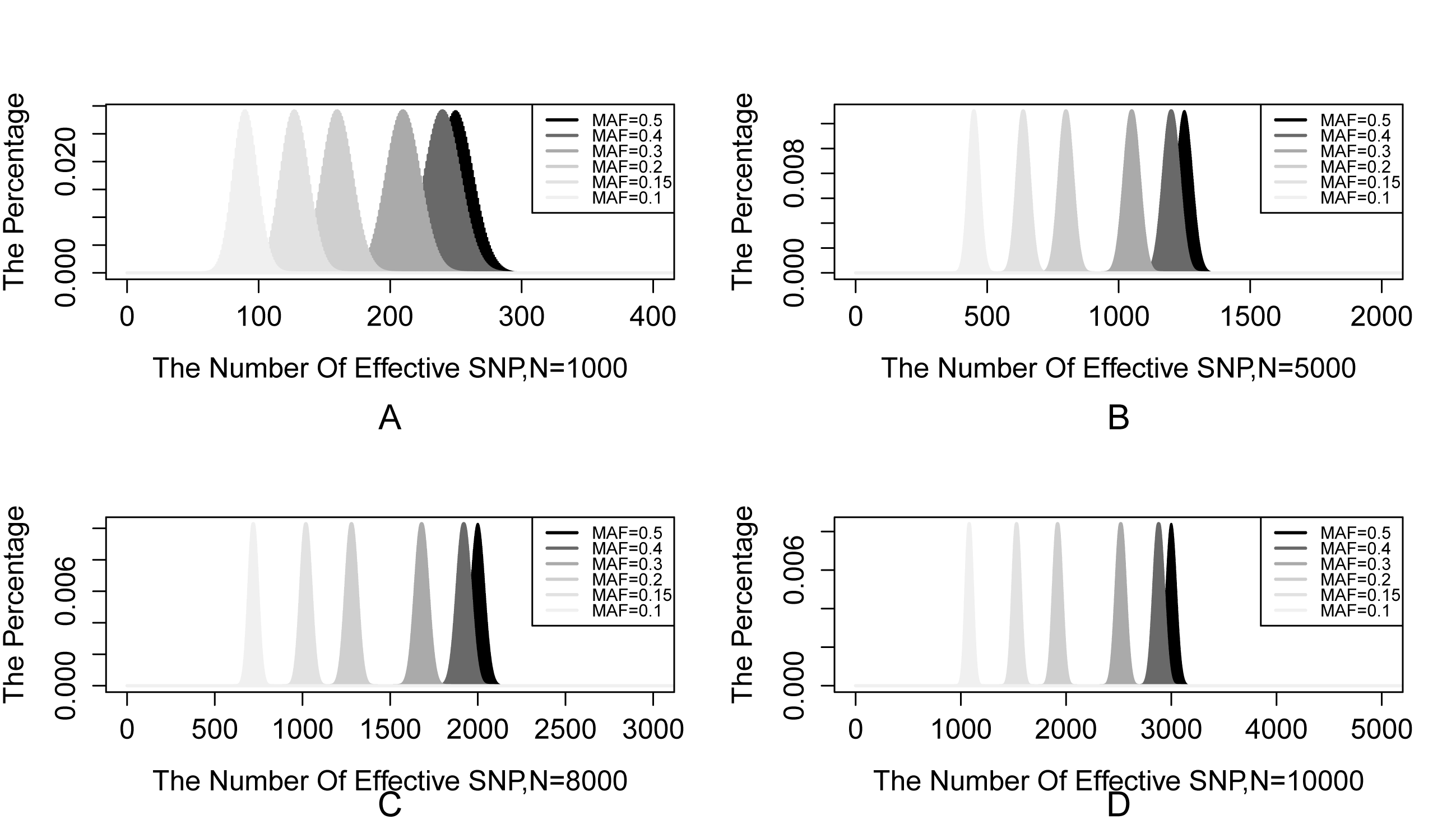

Supplement: S1 Fig — The number of effective SNPs had a positive correlation with the MAF and the number of total SNPs in designed array. (TIF) [file pone.0159385.s001.tif]

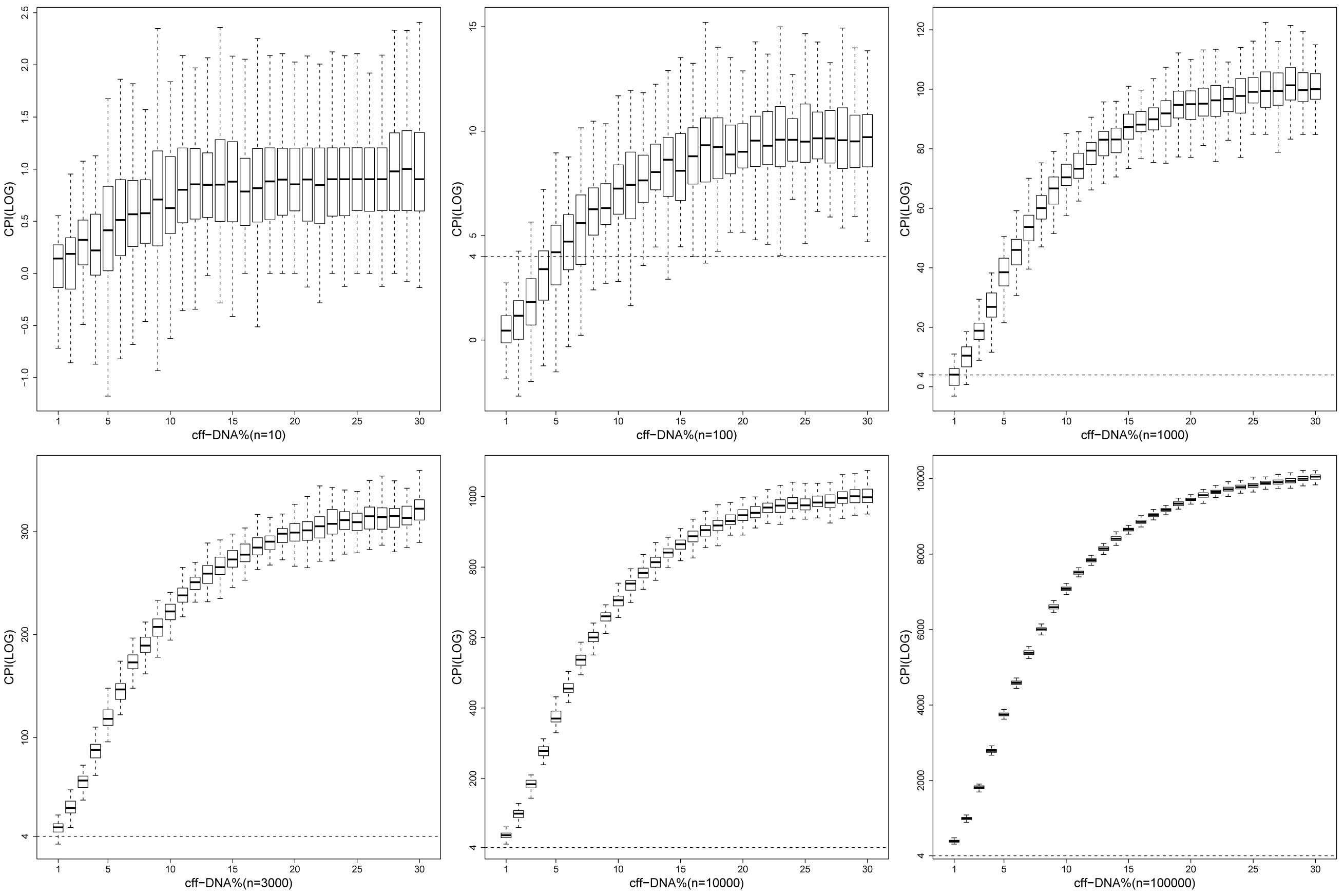

Supplement: S2 Fig — In the boxplots, the y-axis marks the logarithmic value of combined paternity index, and the x-axis stands for the concentration of cffDNA in the plasma. The plots of a, b, c, d, e and f are corresponded to different number of effective loci, which is predicted in the x-axis. (TIF) [file pone.0159385.s002.tif]

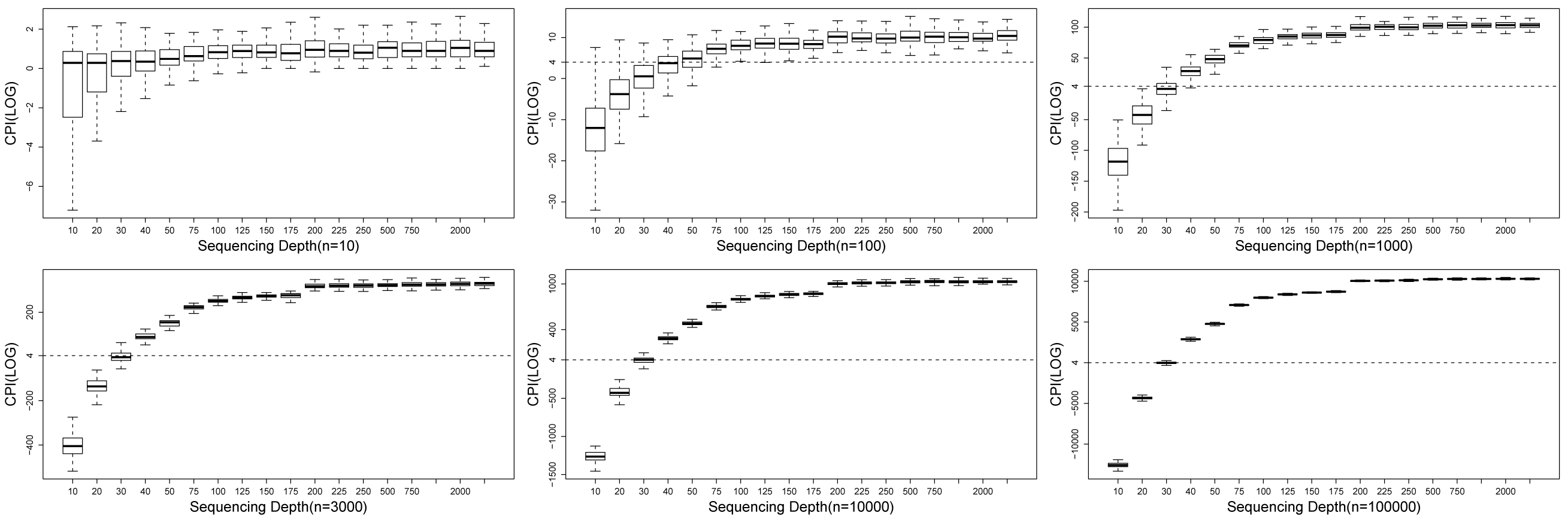

Supplement: S3 Fig — In the boxplots, the y-axis marks the logarithmic value of CPI, and the x-axis stands for the sequencing depth of the plasma. The plots of a, b, c, d, e and f are corresponded to different number of effective loci, which is predicted in the x-axis. (TIF) [file pone.0159385.s003.tif]

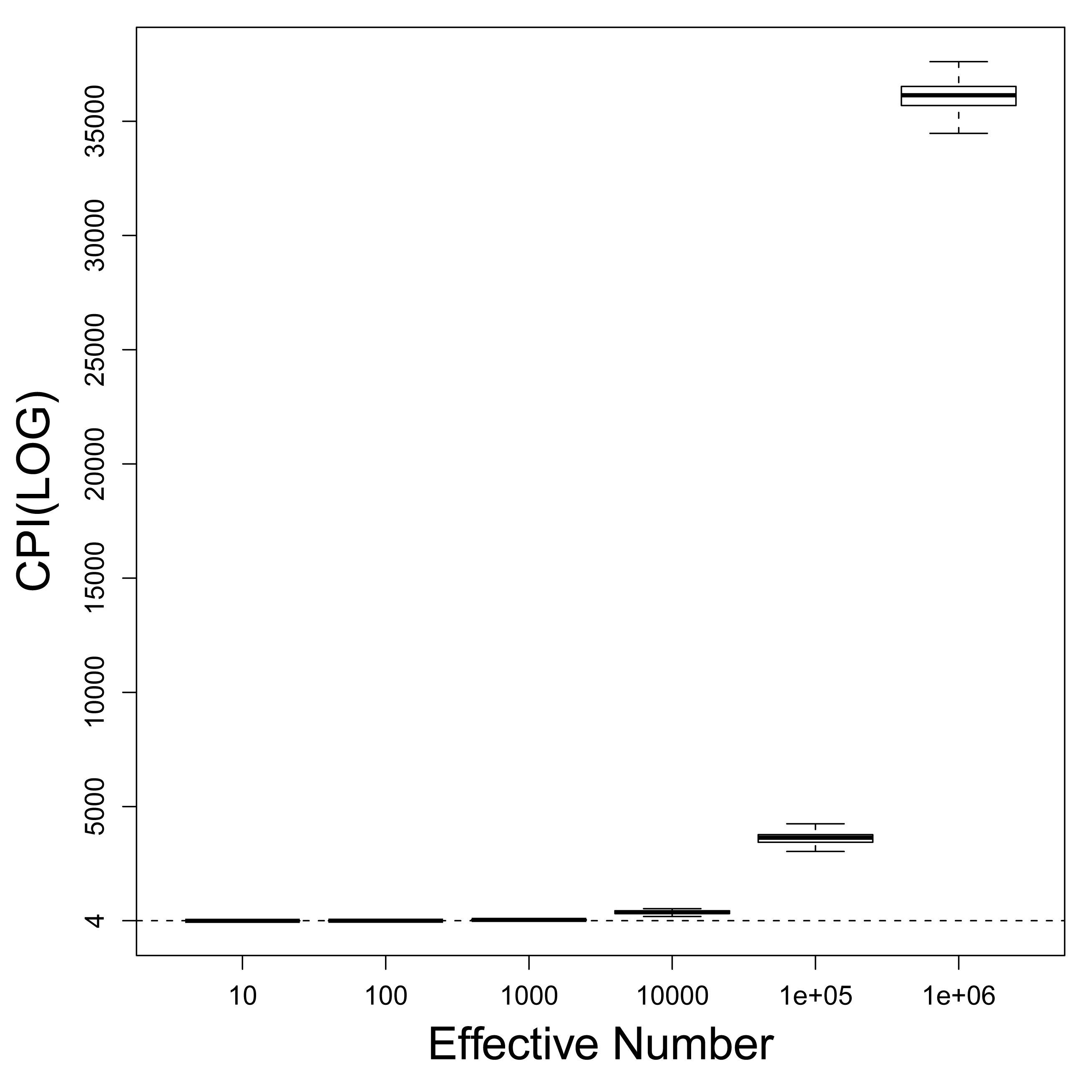

Supplement: S4 Fig — In the boxplots, the y-axis marks the logarithmic value of combined paternity index, and the x-axis stands for the number of effective SNPs. (TIF) [file pone.0159385.s004.tif]
