# Supplementary material for: Noninvasive Prenatal Paternity Testing (NIPAT) through Maternal Plasma DNA Sequencing: A Pilot Study
Source: PLoS One. 2016 Sep 15;11(9):e0159385. doi: 10.1371/journal.pone.0159385 (PMC5025199; doi:10.1371/journal.pone.0159385)
Supplement: S1 File — Detail information of PI calculation formula was listed in the S1 file. (DOCX) [file pone.0159385.s005.docx]

**Detail information of PI formula**

**When the locus was homozygous in mother and fetus (e.g. AA), the distribution of ATCG in plasma was as follows (**$\varepsilon$**stands for fetal fraction,** $\delta$ **stands for error rate):**

| A | T | C | G |
| --- | --- | --- | --- |
| $(1-\varepsilon)(1-\delta)+\varepsilon(1-\delta)$ | $(1-\varepsilon)\frac{\delta}{3}+\frac{\varepsilon\delta}{3}$ | $\frac{\delta}{3}$ | $\frac{\delta}{3}$ |

**When the locus was homozygous (e.g. AA) in mother, but was heterozygote in fetus (e.g. AT), the distribution of ATCG in plasma was as follows**$\varepsilon$**stands for fetal fraction,** $\delta$ **stands for error rate):**

| A | T | C | G |
| --- | --- | --- | --- |
|  |  | $\frac{\delta}{3}$ |  |

$$\Pr(Plasma|Mother,Tested-man,Fetus)=\frac{n!}{a_{A}!a_{T}!a_{C}!a_{G}!}p_{A}^{a_{A}}p_{T}^{a_{T}}p_{C}^{a_{C}}p_{G}^{a_{G}}$$

**PI for one locus:**

$$PI=\frac{X}{Y}=\frac{\Pr(Plasma|Mother,tested-man)}{\Pr(Plasma|Mother,AF)}$$
